# 3.6 mW Active-Electrode ECG/ETI Sensor System Using Wideband Low-Noise Instrumentation Amplifier and High Impedance Balanced Current Driver

**DOI:** 10.3390/s23052536

**Published:** 2023-02-24

**Authors:** Xuan Tien Nguyen, Muhammad Ali, Jong-Wook Lee

**Affiliations:** Information and Communication System-on-Chip (SoC) Research Center, School of Electronics and Information, Kyung Hee University, Yongin 17104, Republic of Korea

**Keywords:** active electrode, bioimpedance, electrocardiogram, preamplifier, integrated circuit

## Abstract

An active electrode (AE) and back-end (BE) integrated system for enhanced electrocardiogram (ECG)/electrode-tissue impedance (ETI) measurement is proposed. The AE consists of a balanced current driver and a preamplifier. To increase the output impedance, the current driver uses a matched current source and sink, which operates under negative feedback. To increase the linear input range, a new source degeneration method is proposed. The preamplifier is realized using a capacitively-coupled instrumentation amplifier (CCIA) with a ripple-reduction loop (RRL). Compared to the traditional Miller compensation, active frequency feedback compensation (AFFC) achieves bandwidth extension using the reduced size of the compensation capacitor. The BE performs three types of signal sensing: ECG, band power (BP), and impedance (IMP) data. The BP channel is used to detect the Q-, R-, and S-wave (QRS) complex in the ECG signal. The IMP channel measures the resistance and reactance of the electrode-tissue. The integrated circuits for the ECG/ETI system are realized in the 180 nm CMOS process and occupy a 1.26 mm^2^ area. The measured results show that the current driver supplies a relatively high current (>600 μA_pp_) and achieves a high output impedance (1 MΩ at 500 kHz). The ETI system can detect resistance and capacitance in the ranges of 10 mΩ–3 kΩ and 100 nF–100 μF, respectively. The ECG/ETI system consumes 3.6 mW using a single 1.8 V supply.

## 1. Introduction

Bioimpedance sensing has been an important research topic for investigating tissue properties, which provides valuable information for diagnosis, physiology, and pathology [1,2,3]. Dry electrodes have gradually replaced wet electrodes owing to the many advantages in personal healthcare and brain-computer interface applications [4,5]. Dry electrodes enable long-term monitoring in a user-friendly manner; however, they are exposed to relatively high variations in the electrode-tissue impedance [6]. This leads to interference in the wires that connect the electrodes to the readout circuit, thus reducing the signal quality. Some designs use analog buffers in the electrode [7]; this approach performs only impedance conversion, and it still places a stringent low-noise performance on the subsequent readout circuits. An alternative solution to this issue is using an active electrode (AE) containing a preamplifier [8]. This approach allows the electrodes to be placed close to the tissue. Noise interference is reduced by the short path from the electrode to the preamplifier, which relieves the noise requirement of the subsequent back-end (BE) circuits.

However, there are several challenges that are required to be addressed for this approach to be fully compatible with the electrocardiogram (ECG)/electrode-tissue impedance (ETI) sensing system [9,10]. The ETI system uses currents with different amplitudes and frequencies, which are injected into the tissue to measure the impedance spectrum. The variations in skin-electrode impedance are required to be resolved using a driver with a high output impedance over the bandwidth [11]. Various current drivers using the discrete designs of the balanced Howland topology have been reported [12,13]. This approach uses a pair of opamps and resistive networks in the feedback paths. The previous work [13] achieves an output impedance of 750 kΩ at 10 kHz, which is reduced to 330 kΩ at 300 kHz and eventually to 70 kΩ at 1 MHz. The maximum output current was approximately 1 mA_pp_. However, the demand for very accurate matching of resistors makes the Howland topology incompatible with the integrated circuit (IC) design. Second, to obtain a high-quality signal, a preamplifier with a low-noise performance is required for AE. Moreover, electrode polarization, which can be up to a few hundred mV, may reduce the preamplifier headroom and saturate the output unless approaches handling the large DC offset are used. Another issue is that these requirements should be realized using low power to extend the battery life for portable applications.

To address the above challenges, in this work, we present a low-power AE-based integrated system for ECG/ETI measurement. To increase the output impedance, the current driver uses a matched current source and sink, operating under negative feedback. A new source degeneration method is proposed to increase the linear input range. The measured results show that the driver supplies a relatively high current (600 μA_pp_) and achieves a high output impedance (1 MΩ at 500 kHz). To meet the stringent requirement of the AE, a preamplifier is designed using a capacitively-coupled instrumentation amplifier (CCIA) and active feedback frequency compensation (AFFC). The proposed CCIA achieves an excellent noise voltage density and 1/*f* noise corner of 65 nV/√Hz and 2.5 Hz, respectively. The back-end (BE) signal processing IC consists of five channels for three types of signal sensing, which are ECG, band power (BP), and impedance (IMP) data. The BP channel is successfully used to detect the Q-, R-, and S-wave (QRS) complex in the ECG signal. The IMP channel can measure the resistance and capacitance in the ranges of 10 mΩ–3 kΩ and 100 nF–100 μF. Characterization of the complete system shows successful detection of the motion artifact while measuring the ECG/ETI.

The paper is organized as follows. Section 2 describes the system architecture. Section 3 presents the design of the AE. Section 4 explains the design of the BE signal processing IC. Section 5 presents the measured results, and Section 6 draws the conclusion.

## 2. System Architecture

**Figure 1:**
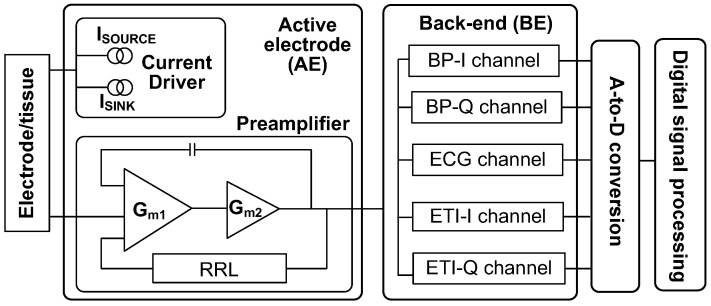
Block diagram for the ECG/ETI measurement system.

Figure 1 shows a block diagram of the proposed ECG/ETI system. The AE consists of a current driver and a preamplifier. The current driver includes matched current source and sink. The preamplifier, which is realized using CCIA, consists of two gain stages (*G_m_*_1_, *G_m_*_2_). The output ripple is suppressed using a ripple-reduction loop (RRL). The BE consists of five channels for three types of signal (ECG, BP, and IMP) sensing. To handle the in-phase and quadrature components, two sub-channels are used for BP and IMP measurement. The QRS complex in the ECG signal is detected using the BP channel. The IMP channel reads out the resistance and reactance, providing information on the electrode-tissue contact conditions. The ECG/ETI system is realized using a one-poly six-metal (1P6M) 180 nm CMOS process. The analog circuits are implemented using thick-oxide (3.3 V) transistors, which are tolerant up to 5 V. The digital circuits are realized using thin-oxide (1.8 V) transistors.

**Figure 2:**
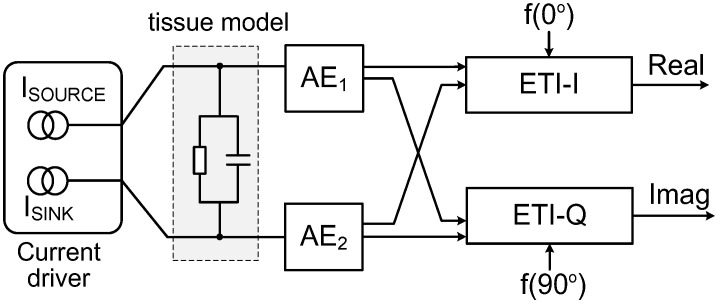
Schematic of electrode-tissue impedance (ETI) measurement.

Figure 2 shows the schematic of the ETI measurement system. The current driver injects a balanced current into the tissue through the electrodes, which can be represented as *i*(*t*) = |*I*|cos(*ωt*), where the angular frequency is *ω* = 2*πf*. The corresponding voltages measured by the active electrodes (AE_1_ and AE_2_) can be written as *v*(*t*) = |*V*|cos(*ωt* + *θ*). Then, we can obtain the in-phase and quadrature components as

(1) $$\begin{array}{ll} v(t) & =|V|\cos\theta \cos(\omega t)-|V|\sin\theta \sin(\omega t)\\ & =R(t)\cos(\omega t)-X(t)\sin(\omega t) \end{array}.$$

The signals are further processed using different phases to obtain resistance *R*(*t*) = |*V*|cos(*θ*)/|*I*| and reactance *X*(*t*) = |*V*|sin(*θ*)/|*I*|. Using the output of the in-phase and quadrature components, the spectral band power *φ*(*f*) of the BP channel can be expressed as

(2) $$\varphi (f)={\left|\int_{-\infty }^{\infty }R(t)w(t)[\cos(2\pi ft)]dt\right|}^{2}+{\left|\int_{-\infty }^{\infty }X(t)w(t)[\sin(2\pi ft)]dt\right|}^{2}$$

where *w*(*t*) is the windowing function [14].

## 3. Active Electrode IC

### 3.1. Current Driver

**Figure 3:**
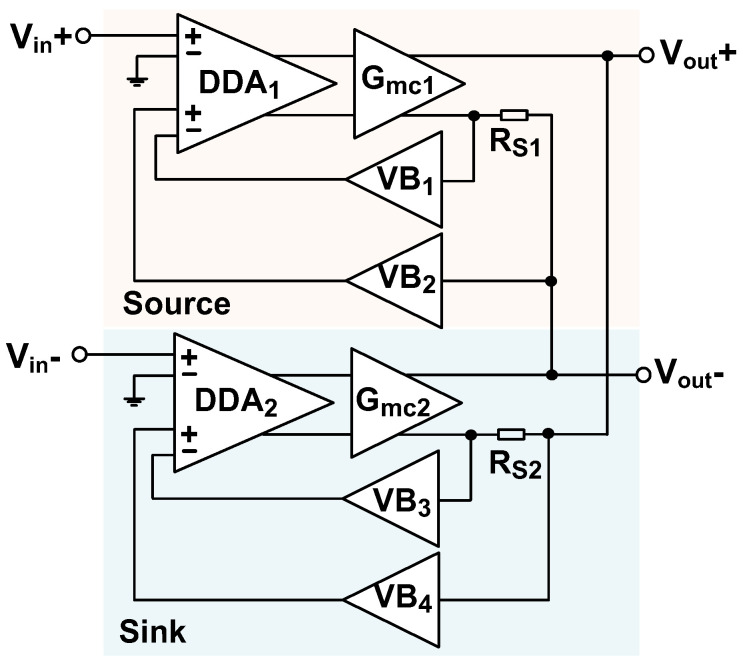
Schematic of the balanced current driver.

Figure 3 shows the schematic of the current driver. It consists of two identical sub-drivers to generate a balanced output. One is used for sinking current, and the other is used for sourcing current. This configuration can reject the common-mode (CM) voltage across the load caused by the mismatch in the transconductor (*G_mc_*_1,2_) and sensing resistors (*R*_S1,S2_). Each sub-driver consists of a differential difference amplifier (DDA_1,2_) followed by the transconductor, which performs the voltage-to-current conversion. The *R*_S1,S2_ is used to monitor the output current, and the voltage across the resistor is fed back to the DDA_1,2_ through a pair of voltage buffers (VB_1,2_ and VB_3,4_), forming a negative-feedback loop. To accommodate a wide output swing, the buffers are designed to achieve a rail-to-rail output [11].

**Figure 4:**
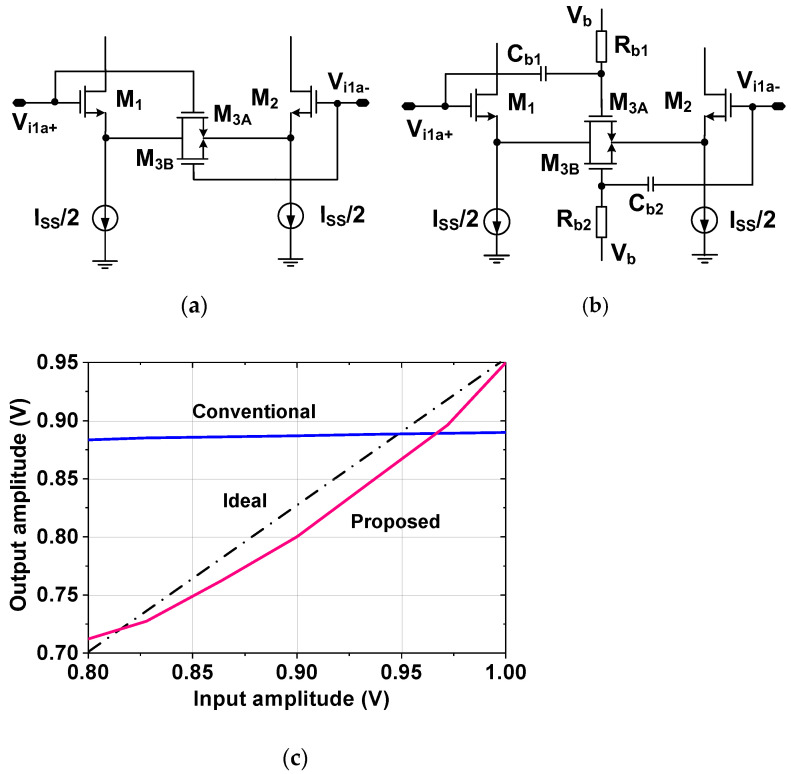
Source degeneration method using (**a**) conventional and (**b**) proposed approach. (**c**) Comparison of the transfer characteristics.

Source degeneration is used to extend the linear range. Figure 4a shows the previous approach of source degeneration [11], where the equivalent resistance of M_3A,3B_ increases the input range. The current sources provide DC biasing to set the quiescent point on which the input AC signal is superimposed. M1 and M2 provide transconductance for the input voltage to current conversion. The current is converted to a voltage by the output resistance. The limitation of this approach is that it requires a relatively large input for M_3A,3B_ to operate in the triode region.

Figure 4b shows the proposed approach where the control voltage of M_3A,3B_ is provided by voltage *V*_b_ through a resistor *R*_b1,b2_. This method allows the source degeneration to be controlled independently of the input level. Additionally, the gate of M_3A,3B_ is connected to the input terminal through a small capacitor *C*_b1,b2_, which allows tracking of the input variations. When the input (*V*_i1a+_, *V*_i1a−_) changes, *C*_b1,b2_ senses the voltage and converts it to current. Then, the current charging the gate capacitance of M_3A,3B_ creates the control voltage, which modulates the on-resistance for source degeneration. Figure 4c shows the comparison of the transfer characteristic obtained using the output and input signal amplitudes, which indicates the improved linearity of the proposed approach.

**Figure 5:**
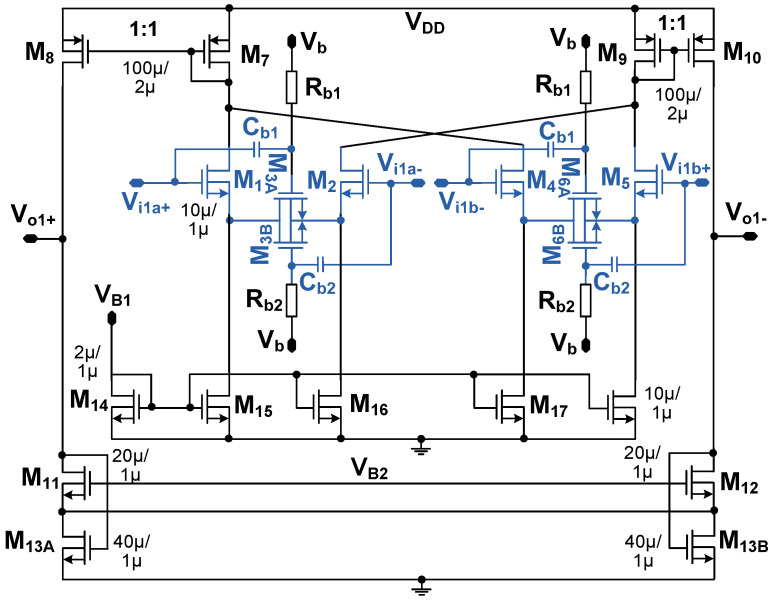
Schematic of the differential difference amplifier. Circuits for source degeneration is shown in blue color. (W/L)_3A,3B_ = (W/L)_6A,6B_ = 1 μm/0.7 μm. *R*_b1,b2_ is realized using a diode-connected transistor having a size of (W/L) = 0.9 μm /0.7 μm. *C*_b1,b2_ = 0.32 pF.

Figure 5 shows the schematic of DDA. M_1,2_ and M_4,5_ form the differential pair for the input transconductor. M_3A,3B_ and M_6A,6B_ provide source degeneration. The currents from the transconductors are summed at the drain of M_7_ and M_9_. M_13A_ and M_13B_ operating in the triode region provide the CM feedback control. The differential output voltage, (*V*_o1+_ − *V*_o1−_), can be written as

(3) $$\begin{array}{ll} V_{o1+}-V_{o1-} & ≅g_{m1,4}(r_{o8}^{}||r_{o11}^{})(V_{i1a+}+V_{i1b-})-g_{m2,5}(r_{o10}^{}||r_{o12}^{})(V_{i1a-}+V_{i1b+})\\ & =g_{m1}(r_{o8}^{}||r_{o11}^{})\left[\left(V_{i1a+}-V_{i1a-})-(V_{i1b+}-V_{i1b-}\right)\right] \end{array}$$

where *g_m_*_i_ and *r*_oi_ represent the transconductance and output resistance of transistor M_i_, respectively.

**Figure 6:**
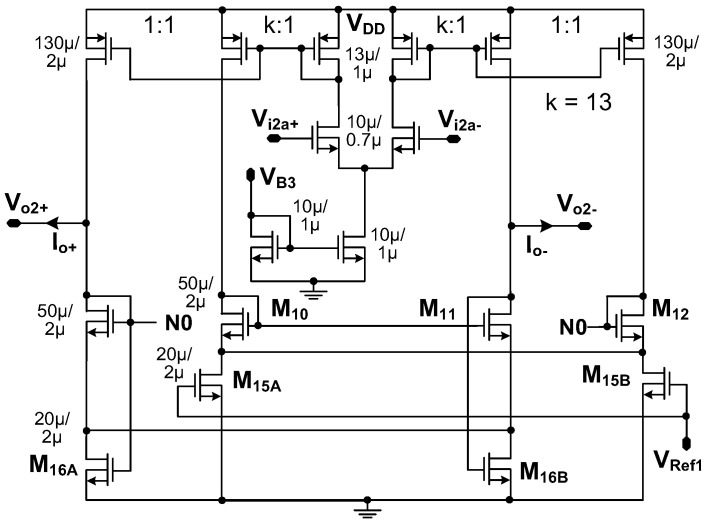
Schematic of the transconductor.

Figure 6 shows the schematic of the transconductor (*G_mc_*_1,2_). It is implemented using the operational transconductance amplifier (OTA) with three current mirrors, which is similar to the one reported in [11]. In this work, the device sizing is modified to be compatible with the supply voltage *V*_DD_ = 1.8 V. The circuit is fully symmetric, and a simple current mirror is used for reduced overdrive voltage. M_16A_ and M_16B_, which operate in the triode mode, are used to set the DC bias. We use *V*_Ref1_ in the secondary current mirror so that M_15A_ and M_15B_ working in the triode region provide additional means of stabilizing the output DC level.

### 3.2. Instrumentation Amplifier

**Figure 7:**
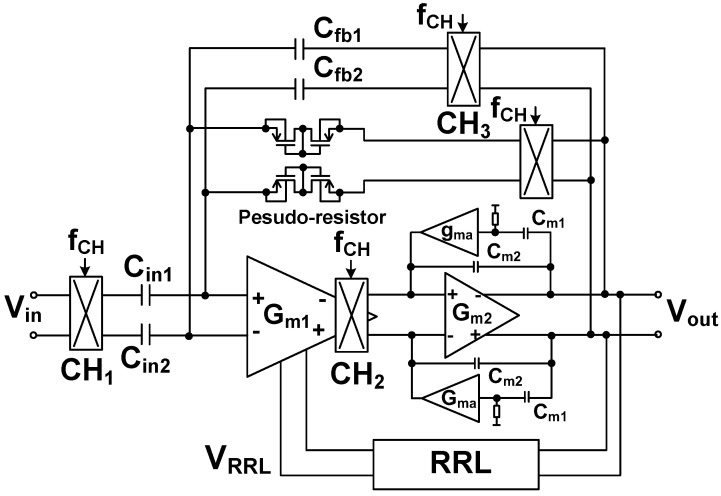
Schematic of the CCIA using active feedback frequency compensation (AFFC) and ripple-reduction loop (RRL). *C*_in1,2_ = 15 pF, *C*_fb1,2_ = 0.14 pF.

Figure 7 shows the schematic of the preamplifier. It consists of two gain stages. The first stage (*G_m_*_1_) is realized using a folded-cascode differential amplifier [15]. The second stage (*G_m_*_2_) is implemented using a common-source amplifier to increase the output swing. Feedback loops are used to set the mid-band gain through *C*_fb1,2_ and the bias of the input node through pseudo-resistors. To suppress the output ripple, the RRL forms another feedback loop. For the RRL, continuous-time implementation is used because the discrete-time approach based on sampling increases the in-band noise through noise folding [16].

The input signal is upconverted by the chopper CH_1_, operating at the chopping frequency *f*_CH_. The signal is amplified by *G_m_*_1_, and the output is downconverted by the chopper CH_2_. The offset at the input of *G_m_*_1_ is upconverted and filtered out by the low-pass characteristic of the amplifier. A capacitive coupling consisting of the input (*C*_in1,2_) and feedback capacitors (*C*_fb1,2_) is built around the two-stage opamp (*G_m_*_1_ and *G_m_*_2_). The combination of CH_1_ and *C*_in1,2_ creates an equivalent input resistance of (1/2*f*_CH_*C*_in1,2_). Similarly, CH_3_ and *C*_fb1,2_ create an equivalent resistance of (1/2*f*_CH_*C*_fb1,2_). The two resistances form a feedback loop around the opamp. 

Nested Miller compensation has been used to achieve stability under a wide range of capacitive loads [15]; however, for stability, the size of the compensation capacitor should be increased in proportion to the load capacitance. Moreover, this approach suffers from bandwidth reduction. In this work, we use AFFC, which has the advantage of extending the bandwidth using a small compensation capacitor. The AFFC is implemented using a cascode Miller (*g_ma_* and *C_m_*_1_) in parallel with a Miller capacitor (*C_m_*_2_) [17]. The cascode Miller, which is implemented using a common-gate transconductor, blocks the feedforward signal that exists in the traditional Miller compensation. Compared to the passive compensation, the gain provided by *g_ma_* reduces the size of *C_m_*_1_. *C_m_*_2_ is used to control the *Q*-factor for stability. The noise power contribution of the AFFC to the input-referred noise is relatively small when divided by (*A*_V1_)^2^, where *A*_V1_ ≈ 60 dB is the voltage gain of the first stage. 

**Figure 8:**
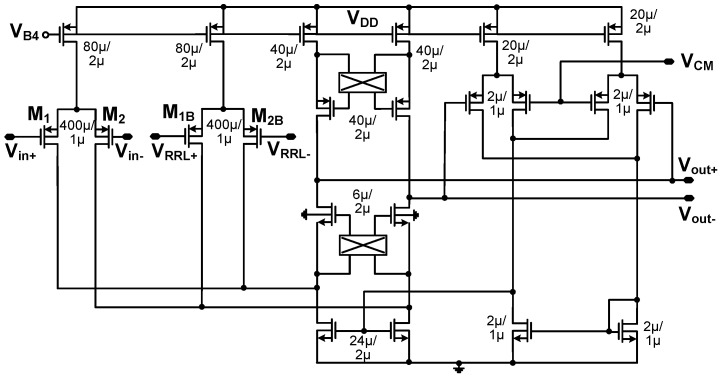
Schematic of the folded-cascode amplifier with common-mode feedback.

Figure 8 shows the schematic of the folded-cascode differential amplifier (*G_m_*_1_). Two additional sets of choppers are embedded in the amplifier. One chopper is placed at the output of the cascode transistors to demodulate the signal down to the baseband while modulating the input offsets to the *f*_CH_ band. Another one is placed at the drain of the current source to upmodulate the flicker noise. At the output of the amplifier, the signal returns to the baseband while the offset and flicker noise is modulated to high frequency, which is filtered by the second stage. The common-mode feedback (CMFB) loop is realized using the two differential pairs. The CMFB loop may have poles of higher frequency than that of the differential-mode (DM) loop. Therefore, we design the CMFB loop to have a smaller unity-gain frequency than the DM loop. Considering the frequency response, we determine the sizing of the two differential pairs. 

**Figure 9:**
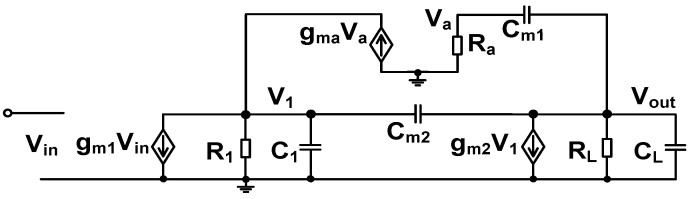
Equivalent small-signal circuit of the amplifier for calculating the open-loop gain.

Figure 9 shows the equivalent small-signal model of the amplifier. Because the open-loop gain is calculated, we obtain the model by removing the feedback loops in Figure 7. It includes transconductances (*g_m_*_1_, *g_m_*_2_, and *g_ma_*) and Miller capacitors (*C_m_*_1_, *C_m_*_2_). *R*_1_ and *C*_1_ are the output resistance and capacitance of the first gain stage, respectively. *R*_1_ is determined by the output resistance of the cascode stage, and *C*_1_ is the sum of capacitance at the output. The *R*_L_ and *C*_L_ are the load resistor and capacitor, respectively. 

Using the circuit model, the open-loop gain of the amplifier can be expressed as.

(4) $$A_{V}(s)≅\frac{A_{\mathrm{DC}}(1+sC_{m1}/g_{ma})g_{ma}(1-sC_{m2}/g_{m2})}{s^{3}R_{1}R_{L}C_{m1}C_{m2}C_{L}+s^{2}R_{1}R_{L}C_{m2}(g_{ma}C_{L}+g_{m2}C_{m1})+sR_{1}R_{L}g_{m2}g_{ma}(C_{m1}+C_{m2})+g_{ma}}$$

where *A*_DC_ = (*g_m_*_1_*g_m_*_2_*R*_1_*R*_L_) is the low-frequency gain. AFFC creates a left-hand plane (LHP) zero, extending the amplifier bandwidth. Assuming the conditions of (*C*_L_, *C_m_*_1_, and *C_m_*_2_) *>> C*_1_, (*g_m_*_1_*R*_1_, *g_m_*_2_*R*_L_) *>>* 1, and (*g_ma_C*_L_) *>>* (*g_m_*_2_*C_m_*_1_), we obtain two zeros (*z*_0_ and *z*_1_) and three poles (*p*_0_, *p*_1_, and *p*_2_) as

(5) $$z_{0}=z_{\mathrm{LHP}}=-\frac{g_{ma}}{C_{m1}}\;z_{1}=z_{\mathrm{RHP}}=\frac{g_{m2}}{C_{m2}},\;p_{0}≅\frac{1}{R_{1}R_{L}g_{m2}(C_{m1}+C_{m2})},\;p_{1}≅-\frac{g_{m2}(C_{m1}+C_{m2})}{C_{m2}C_{L}},\;p_{2}≅-\frac{g_{ma}}{C_{m1}}.$$

The 3-dB frequency is determined by *p*_0_, resulting in the gain-bandwidth product of GBW = *g_m_*_1_/(*C_m_*_1_ + *C_m_*_2_). The right half plane (RHP) zero introduces the phase lag (−tan^−1^(GBW/z_RHP_)) similar to the pole (−tan^−1^(GBW/*p*_0,1,2_)), which reduces the phase margin (PM). The extra phase shift caused by the RHP zero degrades the amplifier stability, and the Miller capacitor (*C_m_*_2_) is used for frequency compensation. 

To realize a maximally flat frequency response for stability, the *Q*-factor can be set to 0.7 [18]. Owing to the positive phase shift provided by LHP zero (z_LHP_), AFFC can extend the bandwidth of the single-stage amplifier by more than two times [19]. Therefore, when *p*_1_ is placed close to twice the value of GBW, the amplifier stability can still be achieved as

(6) $$|p_{1}|≅2\mathrm{GBW}=2\frac{g_{m1}}{\left(C_{m1}+C_{m2}\right)}.$$

To compensate for the negative phase shift caused by *p*_1_, the location of *z*_0_ is selected as

(7) $$\left|z_{0}|=\frac{g_{ma}}{C_{m1}}=\sqrt{2}|p_{1}\right|$$

Using Equations (6) and (7), we obtain the expression for *g_ma_* as

(8) $$g_{ma}=2\sqrt{2}\frac{g_{m1}C_{m1}}{C_{m1}+C_{m2}}$$

To obtain the expression for *C_m_*_1_, we substitute *p*_1_ of Equation (5) into Equation (7). Using the expression for *g_ma_* given by Equation (8), we obtain

(9) $$C_{m1}=\sqrt{\frac{2g_{m1}C_{m2}C_{L}}{g_{m2}}}-C_{m2}$$

where we use relatively small capacitors of *C_m_*_1_ = 0.8 pF and *C_m_*_2_ = 0.2 pF. The *g_ma_* is implemented using a common-gate transistor having a size of (W/L) = 0.9 μm/0.7 μm. The PM of the amplifier with pole-zero cancellation can be expressed as

(10) $$\mathrm{PM}=90{}^{\circ}-\tan^{-1}\left[\frac{\mathrm{GBW}/p_{1,2}}{Q\left(1-{\left(\mathrm{GBW}/p_{1,2}\right)}^{2}\right)}\right]+\tan^{-1}(\frac{\mathrm{GBW}}{z_{0}})$$

where the quality factor is expressed as *Q* = |*p*_1,2_|/(2|Re{*p*_1,2_}|) in case when complex poles are formed for *p*_1_ and *p*_2_ [20]. A detailed derivation of the complex poles and the *Q*-factor can be found in the Appendix A. 

**Figure 10:**
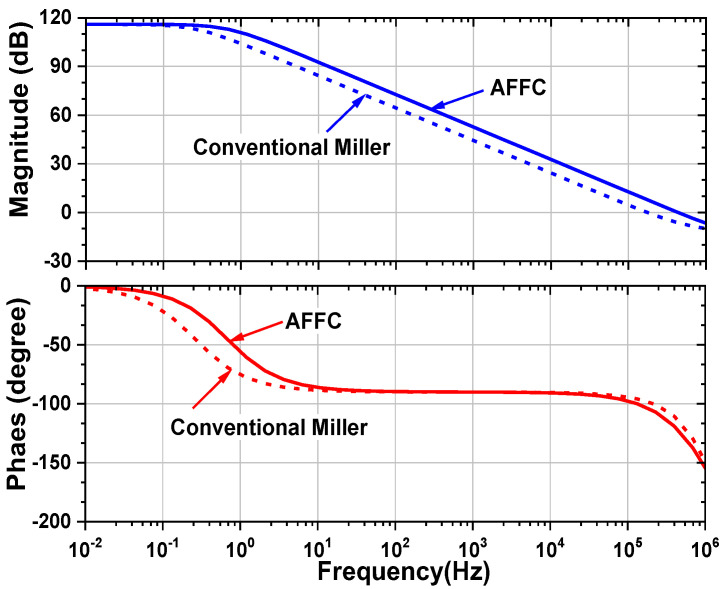
Comparison of the open-loop gain of the amplifier using AFFC and traditional Miller compensation.

**Figure 11:**
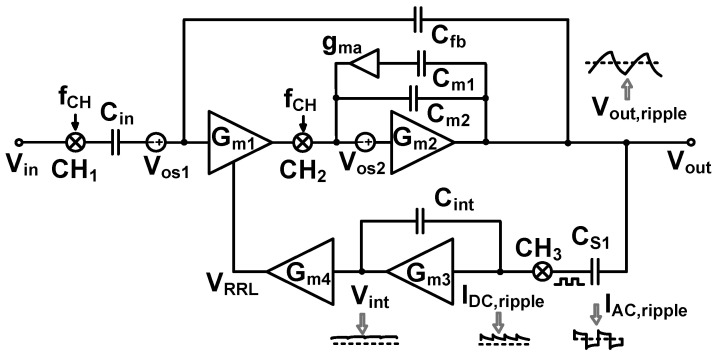
Schematic showing the operation of the ripple-reduction loop.

Figure 10 shows the comparison of the open-loop gain of the amplifier using AFFC and conventional Miller compensation. The bandwidth is extended by the LHP zero available in the AFFC. The PM is reduced from 82.2° to 60°; it guarantees stable operation. Figure 11 explains the operation of the RRL, and its implementation is similar to the previous work [21]. The process variation creates offsets. *G_m_*_1_ and *G_m_*_2_ are associated with the offset voltages *V*_OS1_ and *V*_OS2_, respectively. The *V*_OS2_ is not chopped but suppressed by the open-loop gain (*A*_OL1_) of the preceding stage (*G_m_*_1_). Therefore, the input-referred offset can be written as (*V*_OS1_ + *V*_OS2_/*A*_OL1_). The system offset amplified by *G_m_*_1_ creates the offset current. The current is up-converted to *f*_CH_ by CH_2_, and integrated by *G_m_*_2_ and *C_m_*_2_, producing the output ripple *V*_out,ripple_. At the output, a sense capacitor *C*_S1_ converts *V*_out,ripple_ to a current *I*_AC,ripple_. The current demodulated by the chopper CH_3_ is converted to *I*_DC,ripple_. The current integrated by *G_m_*_3_ and *C*_int_ generates the integrator output *V*_int_, which is converted to the voltage *V*_RRL_ by *G_m_*_4_. To cancel the offset current generated by *V*_OS_, the *V*_RRL_ is input to *G_m_*_1_. 

## 4. Back-End Signal Processing IC

**Figure 12:**
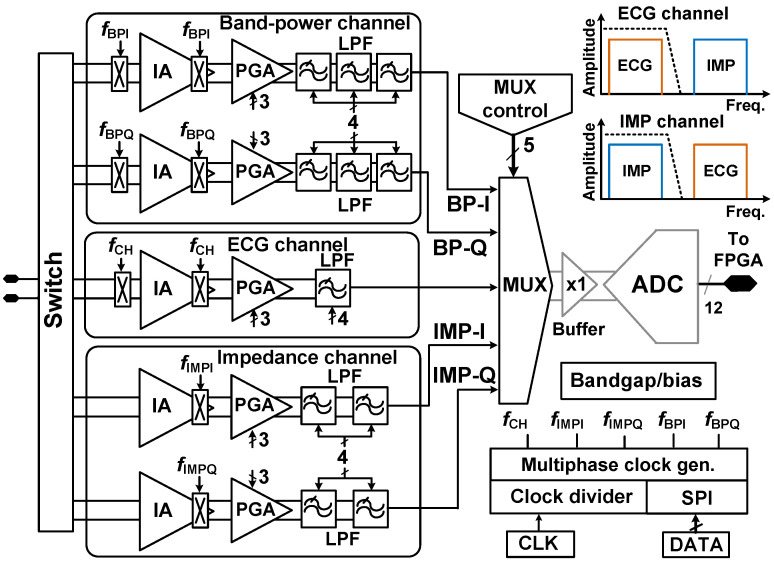
Block diagram of the back-end signal processing IC. The spectrums of the ECG and IMP channels are shown in the inset.

Figure 12 shows a block diagram of the BE signal processing IC. It consists of five channels for three types of measurements (ECG, BP, and IMP). Each channel includes an instrumentation amplifier (IA), a programmable gain amplifier (PGA), and a low-pass filter (LPF). The IC includes a multiphase clock generator, a bias generator/bandgap, and a multiplexer. The bias voltages are generated from the internal bandgap. The multiplexed outputs are digitized using a 12-b analog-to-digital converter (ADC) [22]. The ADC is implemented on a separate test board. The QRS peak detection and BP calculation are performed using a field programmable gate array (FPGA).

The BP channel extracts the information related to the QRS complex using the in-phase and quadrature chopping frequencies of *f*_BPI_ = (*f*_CH_ *+* Δ*f*)(0°) and *f*_BPQ_ = (*f*_CH_ *+* Δ*f*)(90°), respectively. The offset Δ*f* is used to compensate for the channel delay. The IMP channel uses the in-phase and quadrature chopping frequencies of *f*_IMPI_ = *f*_AC_(0°) and *f*_IMPQ_ = *f*_AC_(90°), respectively, where *f*_AC_ is the AC frequency of the current driver. The non-overlapping clocks are generated from the clock divider and multiphase clock generator. Because there is no input chopper in the IMP channel, the ECG signal is upmodulated to *f*_AC_ at the IA output of the IMP channel, which is suppressed by the LPF [8]. If *f*_AC_ = 4 kHz and *f*_CH_ = 2 kHz is chosen, the signal input to the ECG and BP channels experiences two times frequency modulation, and the residual signals at 4 kHz and 8 kHz can be suppressed by the LPF.

**Figure 13:**
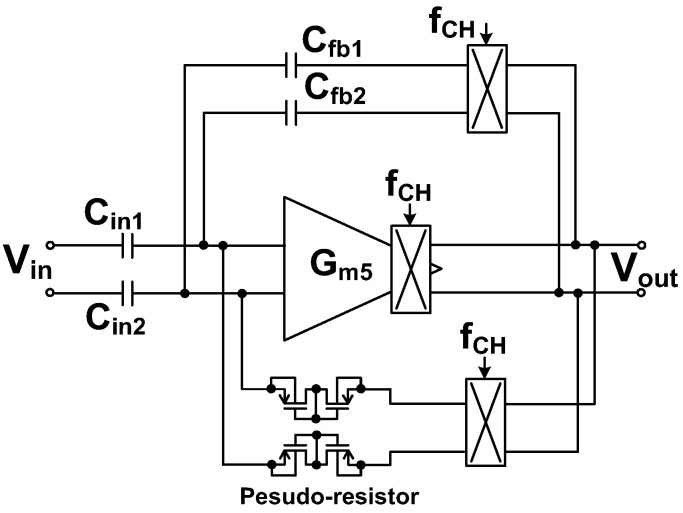
Schematic of the instrumentation amplifier.

Figure 13 shows the schematic of the IA using capacitive coupling. It consists of a folded-cascode amplifier (*G_m_*_5_), re-used from the preamplifier (Figure 8) without the RRL input. The input and feedback capacitors (*C*_in1,2_ and *C*_fb1,2_) determine the closed-loop gain (*C*_in1,2_/*C*_fb1,2_), and the pseudo-resistors define the CM voltage.

**Figure 14:**
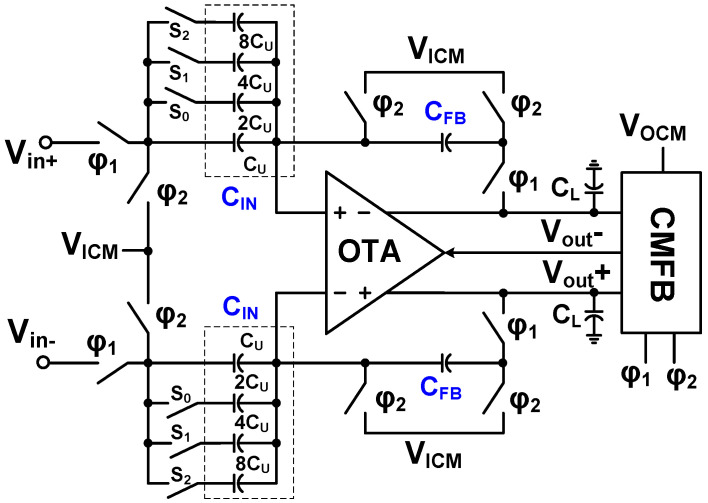
Schematic of the PGA.

Figure 14 shows the schematic of PGA. It consists of an operational transconductance amplifier (OTA), switches, and an array of capacitors. The first stage of the OTA uses a differential amplifier with internal positive feedback for gain boosting [15]. The second stage uses a common-source amplifier for increased output swing. The CMFB is implemented using a switched-capacitor (SC) network clocked by *φ*_1_ and *φ*_2_ [23]. When *φ*_1_ is high (*φ*_2_ is low), the amplifier operates in the negative feedback mode using the input capacitor (*C*_IN_) and the feedback capacitor (*C*_FB_). When *φ*_2_ is high (*φ*_1_ is low), it operates in CMFB mode using the CM input (*V*_ICM_) and the output (*V*_OCM_). The low-frequency closed-loop gain (*A*_CL_) can be expressed as

(11) $$A_{\mathrm{CL}}=\frac{A_{\mathrm{OL}}}{1+(C_{\mathrm{FB}}/C_{\mathrm{IN}})A_{\mathrm{OL}}}≅\frac{C_{\mathrm{IN}}}{C_{\mathrm{FB}}}$$

where *A*_OL_ is the open-loop gain of the OTA. The feedback capacitor is given by *C*_FB_ = *C*_U_ = 100 fF. The *C*_IN_ is the capacitor sum connected to the input, which is determined by closed switches (S_0_–S_2_). The *A*_CL_ is variable from 1 to 15 (*v*/*v*) by controlling the three switches.

**Figure 15:**
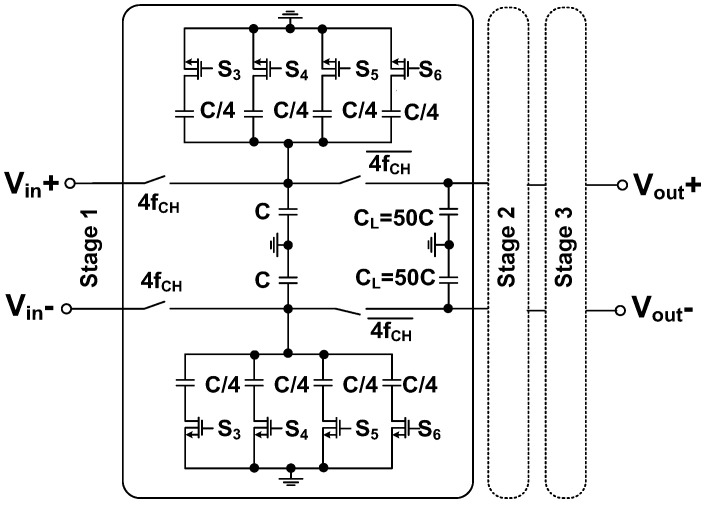
Schematic of the low-pass filter.

**Figure 16:**
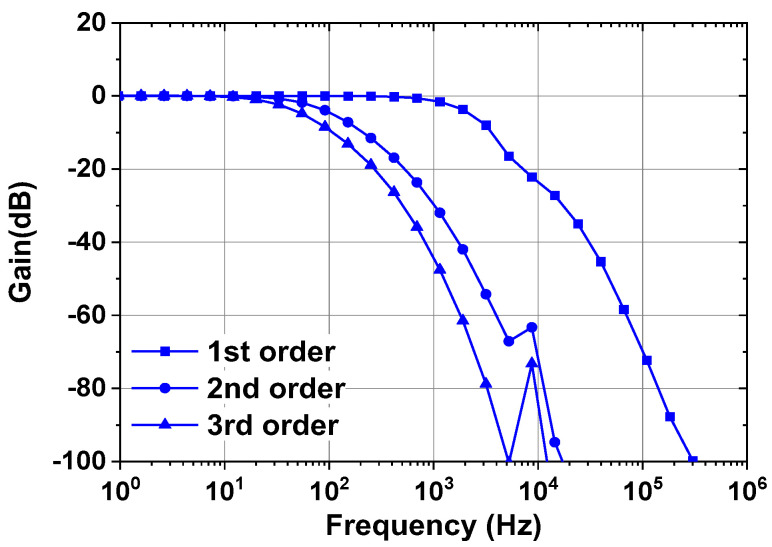
Simulated frequency responses of the low-pass filters.

Figure 15 shows the schematic of the LPF. Using the unit capacitor *C* = 100 fF, the states of the switches (S_3_–S_6_) determine the cutoff frequency. The number of SC stages determines the order of the filter. Figure 16 shows the simulated frequency responses of the LPFs. The ECG channel uses the first-order LPF to set the cutoff frequency close to 1.5 kHz. The IMP and BP channels use the second and third-order LPF to set the cutoff frequency at 60 Hz and 50 Hz, respectively.

## 5. Measured Results

**Figure 17:**
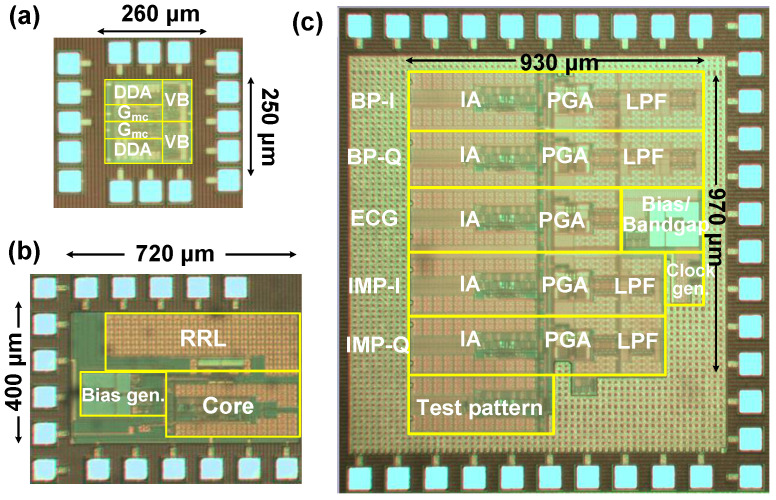
Microphotograph of (**a**) current driver, (**b**) preamplifier, (**c**) back-end signal processing IC.

Figure 17 shows the fabricated ICs for ECG/ETI system using the 180 nm CMOS process. The core size of the current driver and the preamplifier are 0.065 mm^2^ and 0.29 mm^2^, respectively. The core area of the BE signal processing IC is 0.9 mm^2^. The chips are mounted on three test boards for individual characterization using the chip-on-board (COB) technique. After functional testing, they are assembled for the ECG/ETI system characterization.

**Figure 18:**
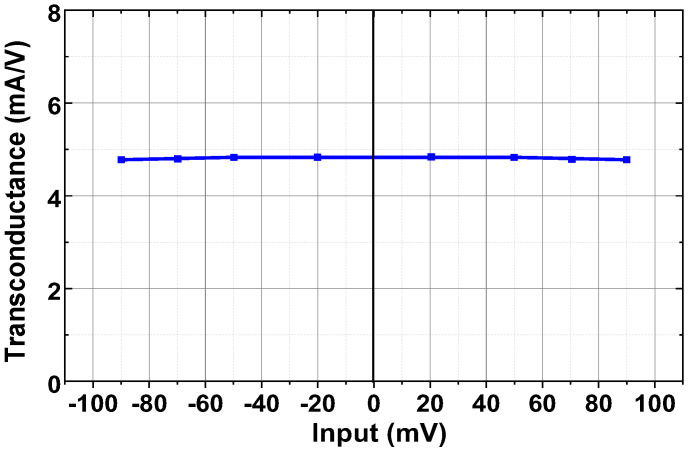
Measure transconductances as a function of input voltage.

**Figure 19:**
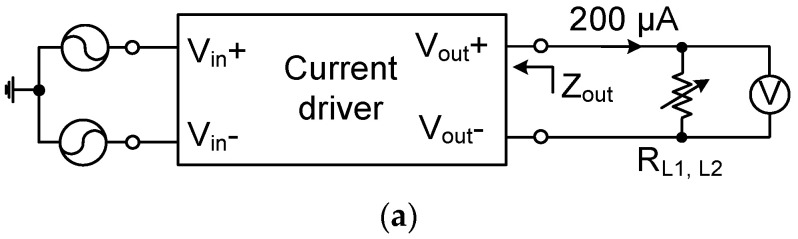

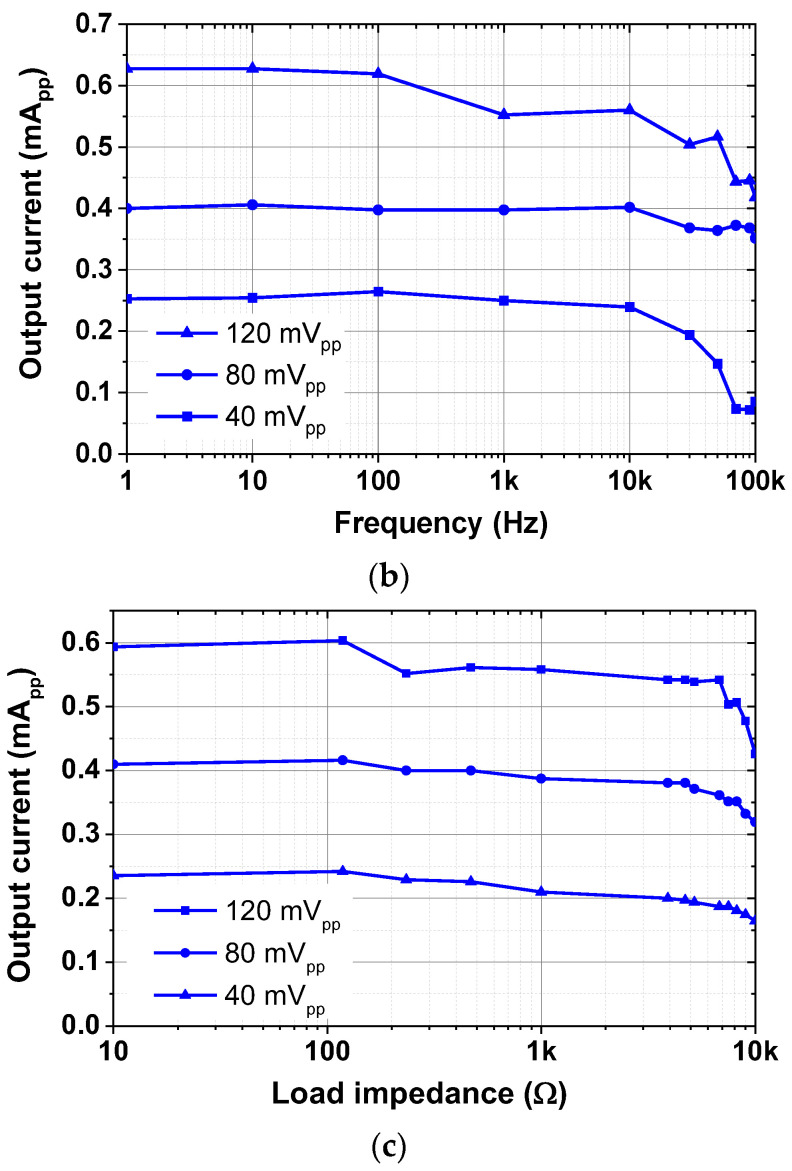
(**a**) Schematic of characterizing the output impedance. (**b**) Measured output current as a function of frequency under different inputs. (**c**) Measured output current as a function of load impedance under different inputs.

Figure 18 shows the measured transconductances of the current driver at 10 kHz using a load of (1 kΩ || 20 pF). The transconductance is relatively constant over the input range from −90 mV to +90 mV. Figure 19a shows the schematic of characterizing the output impedance *Z*_OUT_ of the current driver. The differential input of the driver is generated using the Keysight 33500B with a high stability time base option. The output current is set to 200 μA by adjusting the input. The *Z*_OUT_ value is obtained using two resistances (*R*_L1_ = 100 Ω, *R*_L2_ = 4.7 kΩ) and recording the change in the output voltage. The magnitude of *Z*_OUT_ can be expressed as

(12) $$\left|Z_{\mathrm{OUT}}\right|=\left|\frac{V_{L2}-V_{L1}}{\left(V_{L2}/R_{L2})-(V_{L1}/R_{L1}\right)}\right|$$

where *V*_L1_ and *V*_L2_ are measured voltages using *R*_L1_ and *R*_L2_, respectively [24]. The measured |*Z*_OUT_| is kept constant at 1 MΩ up to 500 kHz, reducing to 300 kΩ at 1 MHz. Figure 19b shows the measured output current as a function of frequency for three inputs. The injected current is measured using the voltage across a 100 Ω resistor, which is in series with the output load of (1 kΩ || 20 pF). For the input of 80 mV_pp_, the output current is relatively constant, up to 10 kHz, with a maximum error of 0.63%. The driver can operate up to 1 MHz, where the current is reduced to 0.13 mV_pp_. Figure 19c shows the output current as a function of load impedance. The data are measured at 100 Hz, and a current up to 600 μA_pp_ can be injected into the low impedance load using 120 mV_pp_.

**Figure 20:**
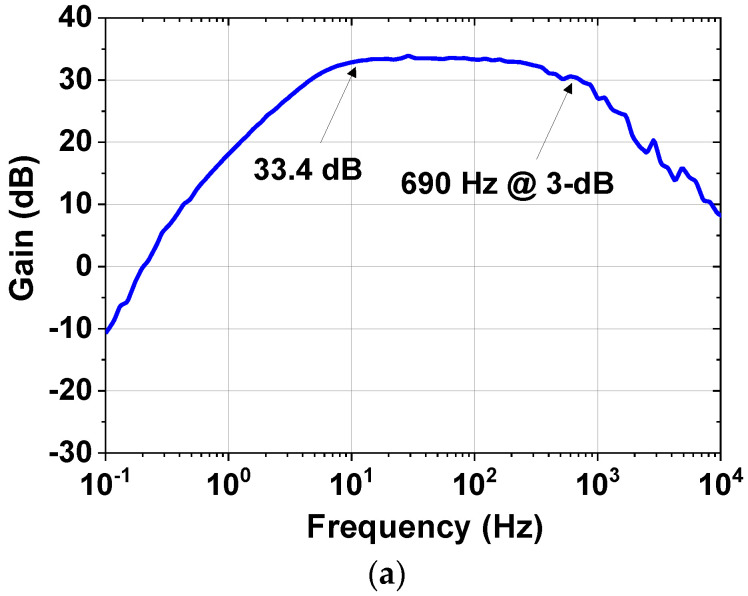

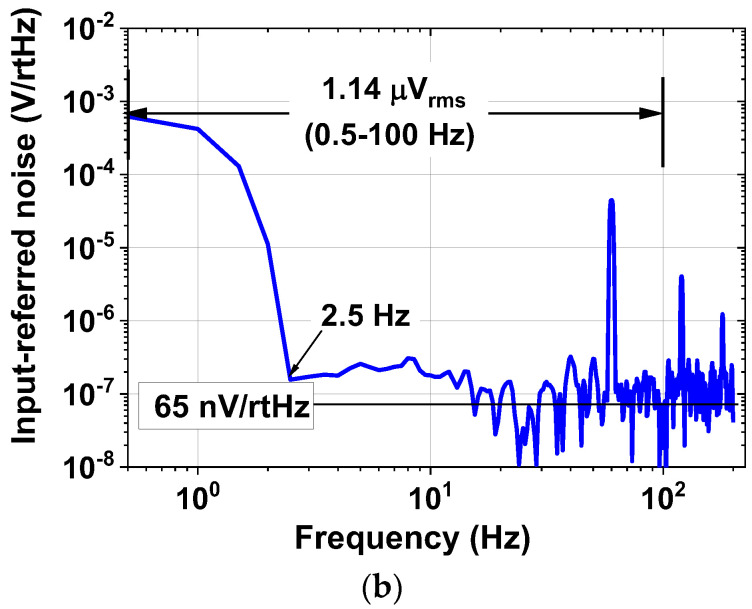
(**a**) Measured gain of the CCIA, (**b**) measured input-referred noise voltage spectral density of the CCIA for the active electrode.

Figure 20a shows the gain response (single-ended) of the preamplifier measured using a Keysight 35670A signal analyzer. The mid-band gain is 39.4 dB (differential) at 100 Hz by consuming 0.56 μA (including bias circuit) from *V*_DD_ = 1.8 V. The mid-band common-mode rejection ratio (CMRR) and power-supply rejection ratio (PSRR) are 61 dB and 66 dB, respectively. Figure 20b shows the measured noise spectral density using *f*_CH_ = 2 kHz. The input noise voltage density is 65 nV/√Hz with a 1/*f* corner frequency of 2.5 Hz. The integrated noise from 0.5 to 100 Hz is 1.14 μV_rms_.

**Figure 21:**
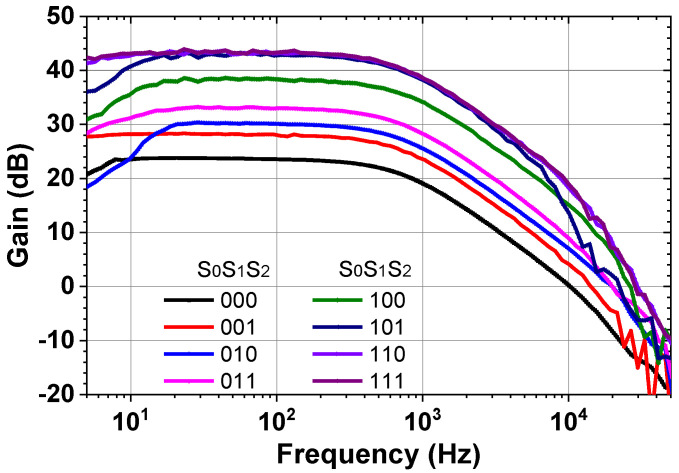
Measured frequency response of the ECG channel for eight gain settings.

**Figure 22:**
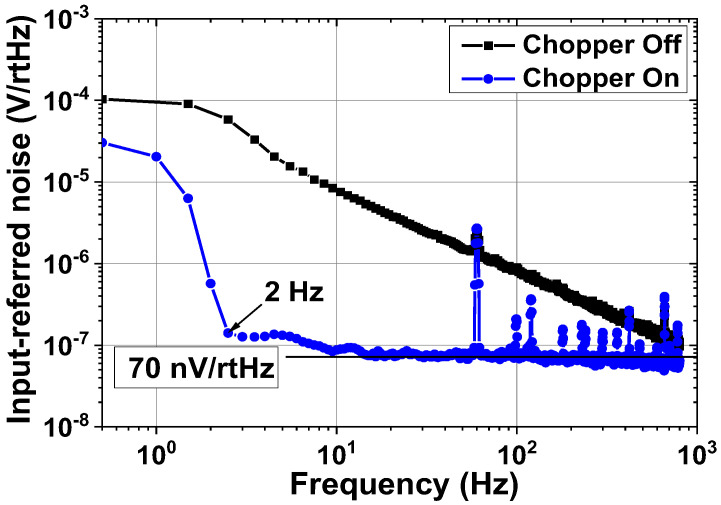
Measured input-referred noise voltage spectral density of the ECG channel.

**Figure 23:**
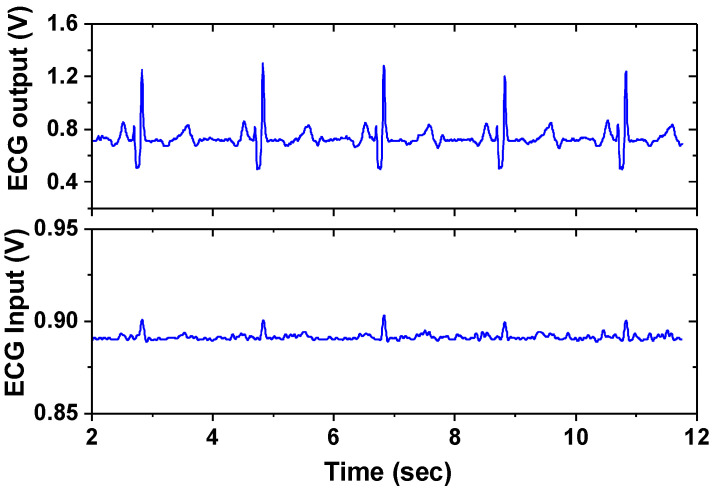
Measured amplified ECG signal.

Next, we present the measured result of BE signal processing IC. Figure 21 shows the measured gain control of the ECG channel. When all the switches of the PGA are in the OFF state, the mid-band gain is 20 dB. When all the switches are in the ON state, the PGA gain is 23 dB, which leads to an overall gain of 43 dB. Figure 22 shows the input noise density of the ECG channel measured using 40 dB gain by consuming 0.4 μA at 1.8 V. When the chopper is turned on, 1/*f* noise is effectively suppressed, resulting in a corner frequency of 2 Hz and noise density of 70 nV/√Hz. Figure 23 shows the amplified ECG signal for the input cardiac signal having *V*_pp_ = 12 mV (differential), generated from a function generator Keysight 33500B. The output shows *V*_pp_ = 600 mV (single-ended), indicating a 40 dB gain.

**Figure 24:**
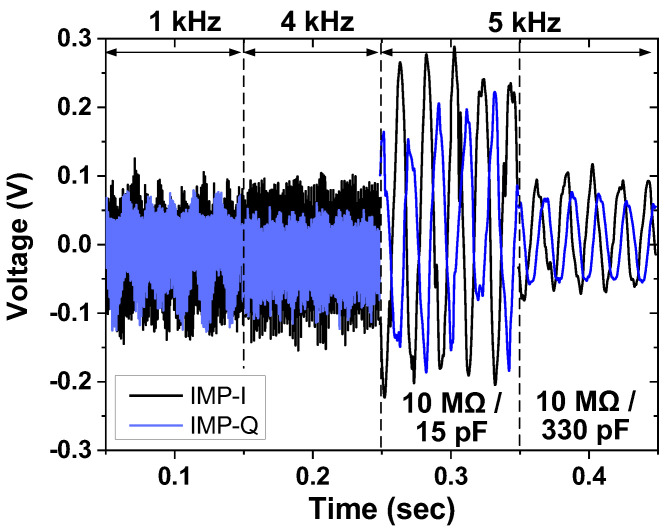
Measured result of the impedance channel. The current driver injects input at three frequencies (1 kHz, 4 kHz, and 5 kHz). The demodulation is performed at 5 kHz.

**Figure 25:**
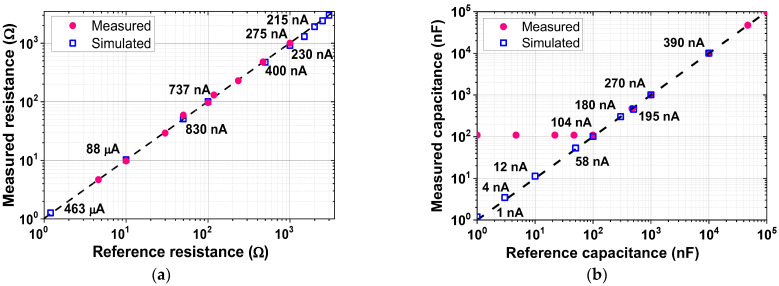
Measured range of the ETI system for (**a**) differential resistance and (**b**) differential capacitance. The values of the injected current are also shown.

Figure 24 shows the measured results of the in-phase and quadrature IMP channels. The result is obtained by injecting current at three frequencies (*f*_AC_ = 1, 4, and 5 kHz). The baseband output is observed when the demodulation chopper frequency is set to 5 kHz. The measured waveforms show that the output amplitude decreases with the increase in the capacitance value from 15 to 330 pF. Figure 25 shows the measured range of the ETI system. A resistor and a capacitor under the test are connected between the inputs of two AEs. When we use a sinusoidal current of 88 μA_pp_ at 1 kHz, the detection range using 69 dB channel gain is from 100 mΩ to 120 Ω. When the injected current is reduced to 215 nA_pp_ with the minimum PGA gain setting, the maximum range can be extended to 3 kΩ. The minimum range is extended to 10 mΩ using the maximum PGA gain. The simulated results agree well with the value of the reference resistance over the range. In the case of capacitance, the detection of the minimum value is limited by the parasitics of wiring capacitance in the test board. The measured linear range of capacitance is from 100 nF to 100 μF.

**Figure 26:**
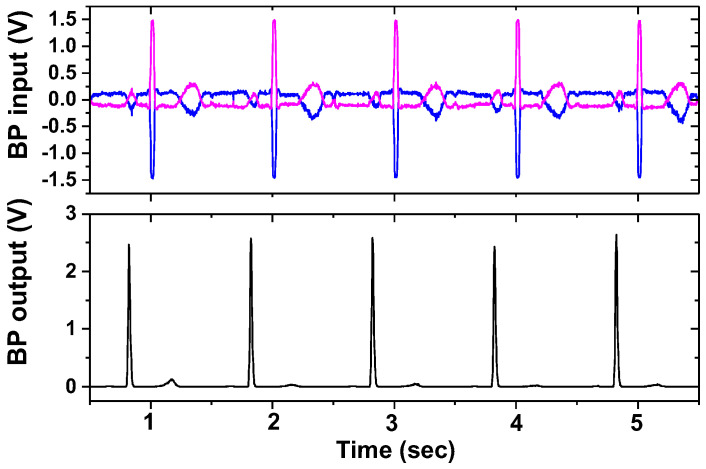
Measured result of band power channels. Positive and negative inputs are indicated with blue and red lines, and output is indicated with black lines.

**Figure 27:**
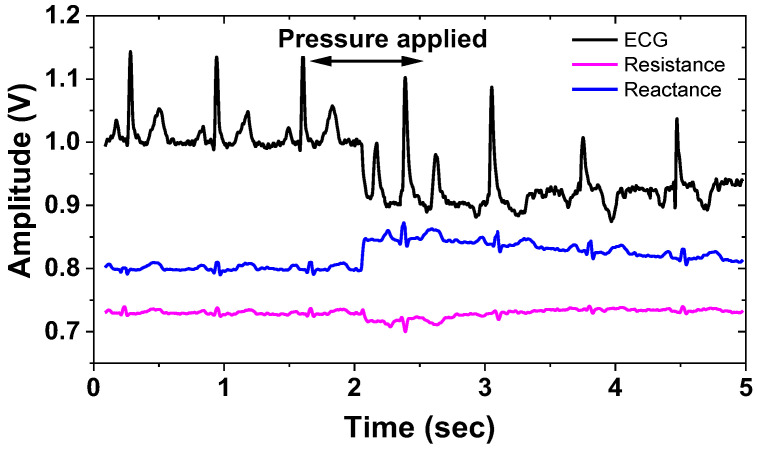
Measured waveforms of the ECG and IMP channels.

Figure 26 shows the measured results of the BP channels using a gain of 43 dB, *f*_CH_ = 2 kHz, and ∆*f* = 50 Hz. The input cardiac signal having *V*_pp_ = 20 mV is generated using a function generator. Figure 27 shows the measured waveforms of the ECG and IMP channels. The electrodes attached to the wrists are connected to the current driver. A differential current of 303 μA_pp_ is injected into the electrode. Other sets of electrodes are connected between the body and test boards (AE and BE), thus amplifying the biopotential signal using 45 dB gain. The environmental noise is suppressed using a filtering function of the Keysight MSO-X oscilloscope. The motion artifact is induced by applying a hard push to the electrode. The result shows a successful operation of the proposed ETI system in vivo, where the ECG signals are measured while ETI is monitored.

**Figure 28:**
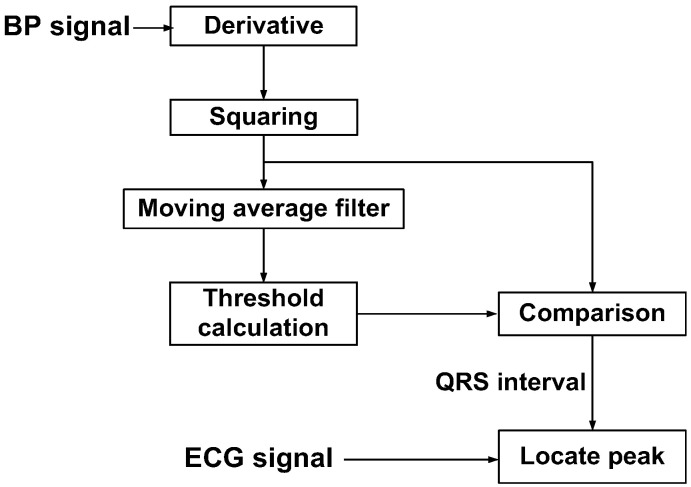
Flowchart of the ECG peak detection algorithm.

**Figure 29:**
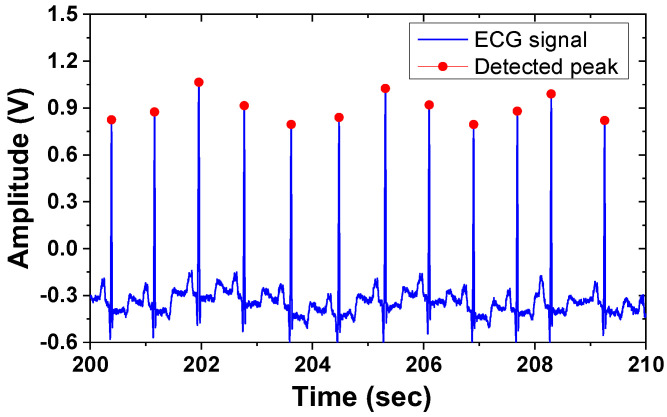
Waveforms of the ECG signal and detected peaks.

Figure 28 shows the block diagram of the peak detection method based on Pan and Tompkins [25]. The magnitude of the signal derivative is processed through a moving-average filter. The detection threshold is set using the measured BP signal. The threshold detects the region of the QRS complexes. Additionally, a time domain search is performed around the detected beat to enhance the accuracy of locating the peak. Figure 29 shows the output of the processed waveform, indicating the successful detection of the ECG peaks. The power breakdown given in Table 1 shows that power consumption is dominated by the current driver. Table 2 shows the performance summary. The proposed ETI system is realized using a 1.26 mm^2^ (excluding ADC) consuming 3.6 mW from a 1.8 V single power supply. 

Table 3 shows a comparison of the current drivers reported in the previous works [11,24,26,27,28]. In [26], electrical impedance tomography (EIT) for hand prosthesis control is proposed for the human-machine interface. The implemented IC uses a fully differential current driver and a current feedback IA, achieving a maximum output current of up to 1 mA_pp_; however, the bandwidth (500 kHz) is lower than ours, and the THD (−42 dB or 0.79%) performance needs further improvement. Work [27] presents a current-conveyor-based current driver for EIT, achieving a wide bandwidth of up to 10 MHz. Targeted for EIT prostate and breast cancer detection, this work shows a high drive current of up to 1.2 mA_pp_; however, the output impedance (101 kΩ) at 1 MHz is lower than ours. The two works [24,27] use a high supply (18 V and 3.3 V), increasing power consumption. Work [28] presents a current driver IC for a portable EIT system, achieving a relatively high output impedance (1 MΩ at 1 MHz) using a 1.2 V supply; however, the maximum output current is limited to 400 μA_pp_ with relatively high THD. The proposed driver features a high output impedance (1 MΩ) and operates at a low supply (1.8 V) suitable for portable system applications. 

Table 4 shows a comparison of the system with other works [29,30,31,32]. Work [29] presents a low-power (0.221 mW) readout IC using a digital-assisted baseline impedance cancellation technique, providing an extended frequency (1 MHz) measurement; however, the impedance range and resolution are lower than ours. In [30], a six-channel EIT system for portable breast cancer detection is proposed and validated in a wearable setting. The system provides a good signal-to-noise (SNR) of up to 90 dB; however, the impedance range is not shown, and it consumes a relatively high power (53.4 mW). Work [31] presents a high input impedance, low noise ETI sensor system using a current mismatch cancellation technique, achieving a relatively good resolution (0.5 mΩ) and low power (0.128 mW); however, the current is limited to 100 μA_pp_ demanding a high gain, high power preamplifier. Work [32] is a commercial system with a balanced impedance range, resolution, and power consumption. Compared to other works, the proposed system achieves the lowest minimum impedance value, a relatively good resolution, and a high driver current. The system is realized using 3.6 mW power suitable for portable applications. 

## 6. Conclusions

This paper presents an active-electrode ECG/ETI sensor system using a wideband low-noise IA and a high-impedance current driver. The balanced current driver uses a matched current source and sink, which operate under negative feedback to increase the output impedance. To increase the linear input range, a new source degeneration method is proposed. The measured output impedance of the driver is 1 MΩ and 300 kΩ at 500 kHz and 1 MHz, respectively. The AFFC technique is applied for the preamplifier to achieve a wide bandwidth using a small Miller compensation capacitor. The proposed IA achieves a noise density and 1/*f* noise corner of 65 nV/√Hz and 2.5 Hz, respectively. The BE signal processing IC is designed for three types of signal monitoring (ECG, BP, and IMP). The measured results show that the output of the BP channel is successfully used for real-time detection of the QRS complex in the ECG signal. Using the gain programmability, the detection range of the IMP channel is increased from 25 to 43 dB. The resistance detection range is from 10 mΩ to 3 kΩ, and the capacitive range is from 100 nF to 100 μF. Characterizing the complete system shows successful detection of the motion artifact while measuring the ECG/ETI using 3.6 mW.

## Figures and Tables

**Table 1:** Power breakdown.

Current driver	1.98 mA @ 1.8 V = 3.56 mW
Preamplifier	0.4 μA (core), 0.16 μA (bias) @ 1.8 V = 1 μW
Back-end	3.7 μA @ 1.8 V= 6.6 μW (ECG: 0.5 μA, ETI: 1.1 μA, BP: 1.1 μA)
Total	3.6 mW

**Table 2:** Performance Summary.

Process		0.18 μm CMOS	
Active electrode	Current driver	Frequency	1 MHz (max)
		Amplitude	600 μApp
		Size	0.065 mm^2^
	Preamplifier	Gain	39.4 dB (differential)
		Input noise density	65 nV/√Hz
		Integrated noise	1.14 μVrms (100 Hz)
		Size	0.29 mm^2^
Back-end		Number of channels	5
		Gain	23–43 dB
		Bandwidth	0.5–1 kHz
		Input noise density	70 nV/√Hz
		Size	0.9 mm^2^

**Table 3:** Comparison of the current driver.

	[11]	[24]	[26]	[27]	[28]	This Work
Bandwidth	1 MHz	500 kHz	500 kHz	10 MHz	1 MHz	1 MHz
Output impedance	1 MΩ @ 500 kHz/ 360 kΩ @ 1 MHz	1 MΩ @ 100 kHz/ 500 kΩ @ 500 kHz	750 kΩ @ 500 kHz	101 kΩ @ 1 MHz/ 19.5 kΩ @ 10 MHz	1 MΩ @1 MHz	1 MΩ @ 500 kHz/ 300 kΩ @ 1 MHz
Max. output current	1 mA_pp_	5 mA_pp_	1 mA_pp_	1.2 mA_pp_	400 μA_pp_	>500 μA_pp_
THD	<0.1% * @ 1 mA_pp_	0.69% @ 5 mA_pp_	0.79% @ 3.97 mA_pp_	0.14% * @ 1.2 mA_pp_	<0.5% @ 400 μA_pp_	0.29% ** @ 303 μA_pp_
Supply	±2.5 V	18 V	±1.65 V	3.3 V	1.2 V	1.8 V

* Increases to 0.68% at 10 MHz. ** Measured using driver current less than maximum value at 50 kHz.

**Table 4:** Comparison of system performance.

	This Work	[29]	[30]	[31]	[32]
Frequency (kHz)	100	1–1000	100	100	150
IMP range (Ω)	0.01–3 k	100–2 k	-	20–4 k	2.8 k
Resolution (mΩ_rms_)	3.5	2–14	4.9	0.5	14.9
Current (μA_pp_)	600	100	400	100	667
Modulation	Sine	Square	Sine	Square	Pseudo-sine
Power (mW)	3.6	0.221	53.4	0.128	2.9

## Appendix A

We consider the denominator of Equation (4) to find the poles as

(A1) $$s^{3}R_{1}R_{L}C_{m1}C_{m2}C_{L}+s^{2}R_{1}R_{L}C_{m2}(C_{L}g_{ma}+C_{m1}g_{m2})+sR_{1}R_{L}g_{m2}g_{ma}(C_{m1}+C_{m2})+g_{ma}=0.$$

Case 1: in the case of ($C_{L}g_{ma}<<C_{m1}g_{m2}$), Equation (A1) can be written as

(A2) $$s^{3}R_{1}R_{L}C_{m1}C_{m2}C_{L}+s^{2}R_{1}R_{L}C_{m2}C_{m1}g_{m2}+sR_{1}R_{L}g_{m2}g_{ma}(C_{m1}+C_{m2})+g_{ma}=0,$$

where the three poles can be expressed as

(A3) $$p_{0}\approx -\frac{d_{0}}{d_{1}}=\frac{1}{R_{1}R_{L}g_{m2}(C_{m1}+C_{m2})},$$

(A4) $$p_{1}\approx -\frac{d_{1}}{d_{2}}=-\frac{R_{1}R_{L}g_{m2}g_{ma}(C_{m1}+C_{m2})}{R_{1}R_{L}C_{m2}C_{m1}g_{m2}}=-\frac{g_{ma}(C_{m1}+C_{m2})}{C_{m2}C_{m1}},$$

(A5) $$p_{2}\approx -\frac{d_{2}}{d_{3}}=-\frac{R_{1}R_{L}C_{m2}C_{m1}g_{m2}}{R_{1}R_{L}C_{m1}C_{m2}C_{L}}=-\frac{g_{m2}}{C_{L}}.$$

Case 2: In the case of ($C_{L}g_{ma}>>C_{m1}g_{m2}$), Equation (A1) can be written as

(A6) $$s^{3}R_{1}R_{L}C_{m1}C_{m2}C_{L}+s^{2}R_{1}R_{L}C_{m2}C_{L}g_{ma}+sR_{1}R_{L}g_{m2}g_{ma}(C_{m1}+C_{m2})+g_{ma}=0.$$

where the three poles can be expressed as

(A7) $$p_{0}\approx -\frac{d_{0}}{d_{1}}=\frac{1}{R_{1}R_{L}g_{m2}(C_{m1}+C_{m2})},$$

(A8) $$p_{1}\approx -\frac{d_{1}}{d_{2}}=-\frac{R_{1}R_{L}g_{m2}g_{ma}(C_{m1}+C_{m2})}{R_{1}R_{L}C_{m2}C_{L}g_{ma}}=-\frac{g_{m2}(C_{m1}+C_{m2})}{C_{m2}C_{L}},$$

(A9) $$p_{2}\approx -\frac{d_{2}}{d_{3}}=-\frac{R_{1}R_{L}C_{m2}C_{L}g_{ma}}{R_{1}R_{L}C_{m1}C_{m2}C_{L}}=-\frac{g_{ma}}{C_{m1}}.$$

The expression of *p*_1_ given by Equation (A8) is proportional to the root mean square of the current. When the load current changes, increasing *p*_1_ can affect the value of *p*_2_, creating complex poles. We assume *|s|>>|p*_1_*|* when considering the non-dominant poles, *p*_1_ and *p*_2_. Then, the approximate values of *p*_1_ and *p*_2_ can be obtained by solving the following equation as

(A10) $$s^{2}R_{1}R_{L}C_{m1}C_{m2}C_{L}+sR_{1}R_{L}C_{m2}C_{L}g_{ma}+R_{1}R_{L}g_{m2}g_{ma}(C_{m1}+C_{m2})=0.$$

The above result can be further simplified as

(A11) $$s^{2}C_{m1}C_{m2}C_{L}+sC_{m2}C_{L}g_{ma}+g_{m2}g_{ma}(C_{m1}+C_{m2})=0.$$

with the determinant of

(A12) $$\Delta =C_{m2}C_{L}g_{ma}[C_{m2}C_{L}g_{ma}-4C_{m1}g_{m2}(C_{m1}+C_{m2})]$$

and we obtain the expression for complex conjugate poles as

(A13) $$p_{1,2}=s=\frac{-C_{m2}C_{L}g_{ma}\pm j\sqrt{C_{m2}C_{L}g_{ma}[C_{m2}C_{L}g_{ma}-4C_{m1}g_{m2}(C_{m1}+C_{m2})]}}{2C_{m1}C_{m2}C_{L}}.$$

The above result can be further simplified as

(A14) $$p_{1,2}=s=-\frac{g_{ma}}{2C_{m1}}\pm j\sqrt{\frac{g_{ma}[C_{m2}C_{L}g_{ma}-4C_{m1}g_{m2}(C_{m1}+C_{m2})]}{4C_{m1}{}^{2}C_{m2}C_{L}}}.$$

The *Q*-factor of the non-dominant poles is obtained as

(A15) Q=|p1,2|2|Re{p1,2}|=14+Cm2CLgma−4Cm1gm2(Cm1+Cm2)4Cm2CLgma

where the magnitude can be expressed as

(A16) $$|p_{1,2}{|}^{}=\sqrt{\frac{g_{ma}{}^{2}}{4C_{m1}{}^{2}}+\frac{g_{ma}[C_{m2}C_{L}g_{ma}-4C_{m1}g_{m2}(C_{m1}+C_{m2})]}{4C_{m1}{}^{2}C_{m2}C_{L}}}.$$
